# Ubiquitous Sensor Networking for Development (USN4D): An Application to Pollution Monitoring

**DOI:** 10.3390/s120100391

**Published:** 2012-01-04

**Authors:** Antoine Bagula, Marco Zennaro, Gordon Inggs, Simon Scott, David Gascon

**Affiliations:** 1 ISAT Laboratory, Department of Computer Science, University of Cape Town, Rondebosch, Cape Town, 7701 RSA, South Africa; 2 Telecommunications / ICT for Develoment Laboratory, The Abdus Salam International Centre for Theoretical Physics, Strada Costieri 11, Trieste 34151, Italy; E-Mail: mzennaro@ictp.it; 3 Department of Electrical Engineering, University of Cape Town, Rondebosch, Cape Town, 7701 RSA, South Africa; E-Mails: gordon.ingss@gmail.com (G.I.); simonscott@berkeley.edu (S.S.); 4 Libelium, 11 C/Maria de Luna, Zaragoza 50018, Spain; E-Mail: d.gascon@libelium.com

**Keywords:** ubiquitous sensor networking, environment monitoring, wireless sensor networks, long distance wireless sensor networks

## Abstract

This paper presents a new Ubiquitous Sensor Network (USN) Architecture to be used in developing countries and reveals its usefulness by highlighting some of its key features. In complement to a previous ITU proposal, our architecture referred to as “Ubiquitous Sensor Network for Development (USN4D)” integrates in its layers features such as opportunistic data dissemination, long distance deployment and localisation of information to meet the requirements of the developing world. Besides describing some of the most important requirements for the sensor equipment to be used in a USN4D setting, we present the main features and experiments conducted using the “WaspNet” as one of the wireless sensor deployment platforms that meets these requirements. Furthermore, building upon “WaspNet” platform, we present an application to Air pollution Monitoring in the city of Cape Town, in South Africa as one of the first steps towards building community wireless sensor networks (CSN) in the developing world using off-the-shelf sensor equipment.

## 1. Introduction

Communication networks have evolved from small islands of closed wired networks into mobile pervasive infrastructures that use the Internet as a common platform for all forms of modern communications. This has increased the appearance of mobile applications, users and services [1] in a ubiquitous computing world where mobile devices such as smart phones are becoming more present with more computational power but less visible due to size. On the other hand, the rapid technological advances in sensor/actuator and Radio Frequency Identification (RFID) devices combined with their miniaturisation and cost reduction have paved the way for a new generation of wireless networks referred to as “Ubiquitous Sensor Networks (USNs)” that build on the “embedding” of small devices into daily life objects and the environment and “networking” these devices to provide different services to different users in a heterogenous environment that involves a myriad of computational devices, applications and systems both mobile and fixed.

Traditional sensor/actuator technology enables USNs deployments in a “1-to-m” model where all the nodes also called “motes”, except the sink node, sense their environment and send the collected information to the sink node. The sink node often referred to as “base station (BS)” passes this information to a gateway to which it is connected for further processing. Using this model, WSNs use a mesh-like multi-hop model allowing these networks to (1) span distances much larger than the transmission range of a single node through localised communication between neighbour nodes (2) adapt to network changes, for example, by routing around a failed node using a different path in order to improve performance and (3) use less transmitter power as a result of the shorter distance transmission mode enabled by the potential to achieve localised communication. A new generation of USNs is emerging where the integration of the GSM/GPRS and Bluetooth into the sensor infrastructure enables an “n-to-m” information dissemination model where besides being able to query and be queried by other nodes as well as exchange its data with these nodes, any node of the network may play the role of a sink capable of transmitting its information to remote processing places. Such integration combined with IPv6 addressing for sensor motes have spearheaded the birth of a new first mile of the Internet referred to as the “Internet-of-the-Things (IoT)” where the information is expected to be accessed not only “anywhere” and “anytime” but also using “anything” by “anyone”.

USNs provide unprecedented abilities to identify, observe and understand large-scale, real-world phenomena at a fine spatial-temporal resolution. Their application in developing countries can help bridge the scientific divide [2] and solve problems that affect communities. When deployed as community sensor networks (CSNs), they can help in (1) providing early warnings for natural disasters such as floods, hurricanes, droughts, earthquakes, epidemics, *etc*.; (2) disseminating surveillance information for cities in parks, hotels, forests, *etc*. to support municipality service delivery; and (3) providing enjoyment of the city citizens and tourists through public services support such as monitoring of water quality to ensure that citizens always have clean water or providing free environmental information on the main tourist destinations. However, while USNs infrastructures are experiencing a rapid and wide expansion in the developed countries, their deployment in the developing world is still in its infancy as revealed by only a few experimental deployments which do not scale to cities or regions and sometimes do not even address the real issues faced by local communities. Furthermore, although the USN requirements for deployments in the developed regions might be similar to those of the developing world (*i.e.*, low cost, long distance), the underlying networks are deployed in a different context. For example, the choice between a GSM-based and Zigbee-based communication for sensor reading dissemination is a pertinent difference in this case, as this relates to telecommunication provision in different contexts. Using a low-power and robust Zigbee-based system in a developing context in favor of a costly and less stable GSM-based system, for example, is an outcome that could be conceivably different for developed nations which have a better cellular infrastructure. Many other important parameters such as the cost of acquisition of the WSN equipment, its maintenance and the openness of the WSN technology should be factored differently when designing a USN in the developing country as compared to one in the developed world as they may have different impacts on the wireless sensor deployment.

Besides describing some of the most important requirements for sensor deployment in the developing world, this paper proposes a new USN architecture referred to as Ubiquitous sensor network for development (USN4D) and validates its usefulness through an air pollution monitoring application. Some of its key features that meet the requirements of the developing world include opportunistic data dissemination, multi-layer networking for long distance deployment and interoperability and localisation of information. Building upon the USN4D architecture, we propose a USN deployment platform called “WaspNet” and describe some of the features and experiments conducted using this platform to assess its readiness for field deployment. Finally, we present experimental results obtained from monitoring air pollution in the city of Cape Town in South Africa as a first step towards building CSNs in the developing world using off-the-shelf sensor equipment.

The remainder of this paper is organised as follows. Section 2 presents the requirements for USN4D while Section 3 describes our proposed architecture and highlights some of its main features. Section 4 presents the “WaspNet” development platform and evaluate its readiness for field deployment in terms of long distance deployment and security through encryption. Section 4.5.4 presents an application to pollution monitoring in the city of Cape Town while Section 5 concludes our paper and reports on future work.

## 2. Requirements for USN4D

Some of the requirements for current USNs to be rapidly/widely deployed in Developing Countries include

- **Lower cost of deployment:** Sensor networks are meant to be deployed in thousands of nodes to achieve wider coverage and efficient dissemination of the data traffic from sensor devices to the sink of the network. However, the cost of current generation sensor motes lies in the 100–800 Euros range or even more depending on the type of sensors used by the mote. This cost spans from low (when sensing Temperature, Humidity, Pressure/Weight, Luminosity, Presence (PIR), Stretch, Bend, Vibration, Impact, and Tilt) to medium (in the case of Carbon Monoxide, Methane CH_4_, Hydrogen H_2_, Liquid Presence, Liquid Level, and Hall Effect sensing) and high (when sensing Oxygen O_2_, Ammonia NH_3_, Nitrogen Dioxide NO_2_).
- **Long distance deployment:** While developed countries typically have well developed WiFi/cable network infrastructures, which can be used for long distance dissemination of the information collected by sensors, the lack of existing/reliable infrastructure in the developing world leads to the sensor networks themselves to be used for the long hops. One of the limitations of wireless sensors lies in the communication range. In many practical deployments in the developing countries, applications such as farming or water quality monitoring may require sensor monitoring over long distances of environmental conditions such as temperature, soil moisture and other levels of water troughs at widely separated locations. In such applications, the short wireless range, typically 100–300 m of maximum range, provided by USNs may be become a limiting factor in terms of both cost since multi-hop routing over longer distances may require many sensors and coverage as the short range sensors can cover only a few square meters.
- **Sensor Interoperability:** While funding for WSN projects is usually readily available in the developed countries, funding to purchase new sensors or replace existing sensors with new ones in the developing countries is often an issue that may hinder WSN projects. The deployment of an heterogenous sensor network that mixes and matches old sensors with new ones and/or sensor from different vendors is a feature that can boost wide application of the WSN technology. Current generation sensors are produced by different manufacturers with different proprietary data formats, communication protocols, programming languages, and Application Processing Interfaces (APIs). This makes interoperability a very challenging task and the handling or use of different types of sensors within a single monitoring application a daunting endeavour. While third party solutions have been proposed under the “SensorWeb” umbrella to support interoperable treatment of various sensors in a single monitoring system, caution must be taken about these solutions when intended to be deployed in developing countries as they are usually only software-based, discounting the hardware compatibility. Deployments in the developing world requires both hardware and software interoperability to avoid a high maintenance cost and the international shipping costs to/from the sensor equipment vendors.
- **Wireless Sensor openness:** Open Wireless Sensors are based on the Open Source Software and Open Source Hardware paradigms [3–5] allowing the code used to program them and the information about the hardware design to be freely released. While Open Source Software (OSS) allows the human-readable source code to be made available under a copyright license (or arrangement such as the public domain) that meets the Open Source Definition, Open Source Hardware (OSH) refers to computer and electronic hardware that is designed in the same fashion as Open Source Software. While Open Source Hardware is part of the open source culture that takes the open source ideas to fields other than software, both features are key requirements for USN deployment in developing countries.
- **Field deployment Readiness:** Data quality, credibility, and feedback are key parameters that determine how ready a USN platform is for field deployment. Sensor data can be acquired on different timescales, triggered by different actions, and delivered in a multitude of different ways. For example, sensor data may be collected periodically, randomly, or non-uniformly. Sensor data acquisition, and especially GPS position fixes, may be triggered in response to detected motion rather than continuously, to avoid battery drain and extend lifetime. Similarly, sensor readings may be delivered via Bluetooth, 802.15.4, or GPRS radios, depending on either user interest or data entropy. Many sensor platforms are not yet mature for field deployment in the harsh environmental conditions of the developing countries.
- **Efficient Middleware Designs:** A middleware is a key component of a USN that resides between applications, operating systems, hardware and communication platforms to let the different applications and systems work together. As currently designed, wireless sensor Middlewares are tightly coupled to the particular sensor application that uses the sensor technology. Such situation raises issues as the use and number of WSNs spreads. The design of generic Middleware systems which fulfil the required management roles in WSNs and implement localisation to adapt the monitoring process to local languages, customs, policies and conditions will be a major milestone in the adoption and extension of USN in the developing world.

## 3. Ubiquitous Sensor Network for Development (USN4D)

A common vision of ubiquitous networking consists of launching small, inexpensive, and robustly inter-networked processing devices into distinctly common-place ends such as homes, markets, hospitals, streets, farms, rivers, forests, lakes, rivers, road sides, and workplaces. These devices are distributed at all scales to deliver different services to different users in a heterogeneous environment that involves a number of applications, protocols, operating systems, processors, and architectures.

**Figure 1.**
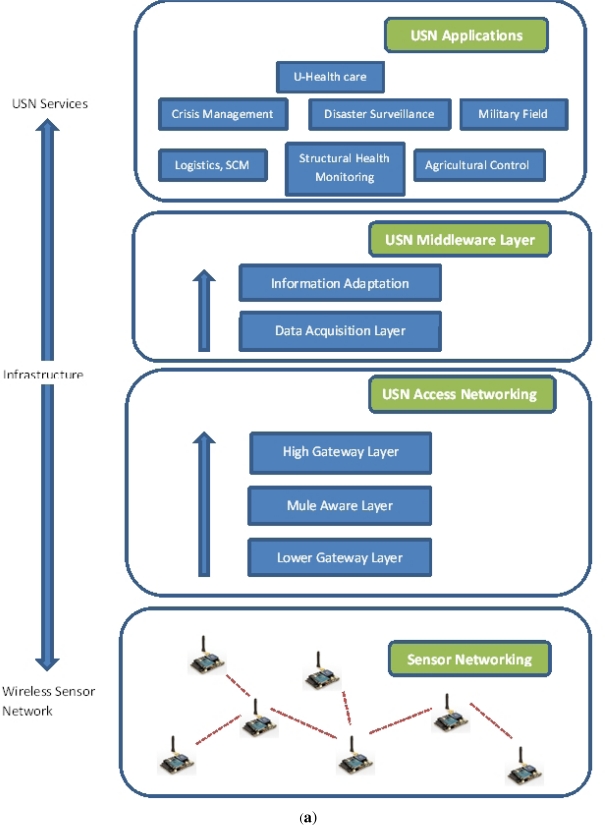

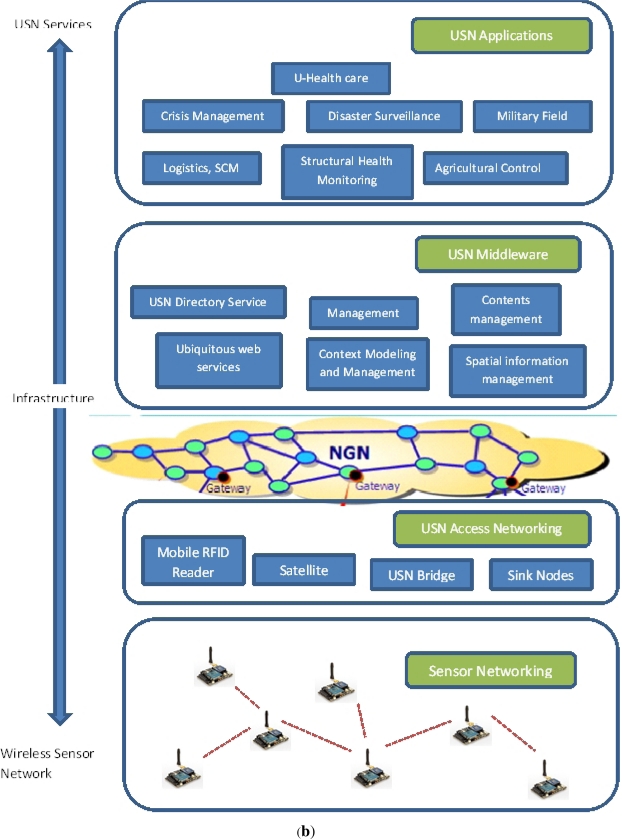
The USN4D Architecture. (**a**) The proposed USN4D architecture; (**b**) The ITU-T USN architecture.

Figure 1(a) illustrates the schematic layers of a USN architecture proposed by ITU-T [6] while Figure 1(b) reveals the different features of our proposed USN4D architecture. In both architectures, four different layers are used to provide different services to different types of applications in a multi-technology, multi-devices and multi-protocol platform. They reveal (1) a sensor networking layer (the bottom layer) where sensor and RFID devices are launched into the environment to sense what is happening and report to sink nodes via USN-bridges (2) A USN access networking layer (the second layer) where the combination of USN-bridges and sink nodes are used as an access network for the first-mile connectivity of a Next Generation Network (NGN) of gateways (3) a USN middleware layer (third layer) used as an interface between the USN access network and the applications layer and (4) different applications which are embodied into a USN applications layer (the last layer) to perform tasks related to logistics, structural health monitoring, agriculture control, disaster surveillance, military field surveillance and disaster/crisis management.

Any ubiquitous networking scenario derived from the architecture depicted by Figure 1(a) uses a communication platform using (1) different protocols such WiFi, wireless LAN and PAN technology, code-division and time-division multiple access wireless communication protocols (2) different devices which are based on different processors such various types of PDAs, smart phones and laptops and (3) all these protocols and devices being built around different architectures such as centralised, distributed or peer-to-peer. In complement to the USN architecture depicted by Figure 1(a), the USN4D architecture behind Figure 1(b) includes (1) a multi-layer USN access network with lower, mule-aware and higher gateway sublayers to support delay-tolerant opportunistic dissemination of the sensor readings (2) a multi-layer Middleware layer supporting sensor system adaptations such as translation from voltage to number in a data acquisition sublayer and local adaptations such as speech conversion from English to a local language in an information localisation sublayer and (3) different other key features embedded in the different layers of the USN4D architecture as described below.

### 3.1. Long-Range Deployment and Inter-Operability

**Figure 2.**
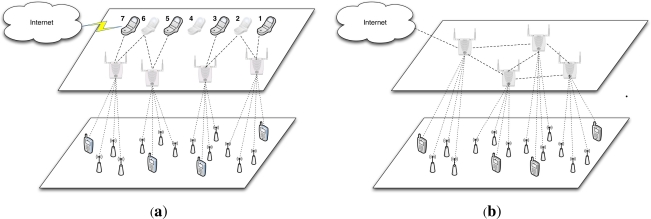
Long-range Deployment and Interoperability. (**a**) Multi Star with Mobile Sink; (**b**) Multi Star with Meshed Sink.

Long distance USN deployment is a key requirement for USN4D that may be met using (1) sensor power increase at the expense of USN lifetime (2) external antenna use and alignment at the expense of flexibility and indoor restrictions and (3) lower frequency use at the risk of country frequency allocation policies restrictions. As flat USN architectures are range-limited, we propose a multi-layer model called “gateway networking” that extends the range of a USN by layering a network of gateway devices above islands of sensor motes to build star topologies below mesh or star gateway networks. Besides reducing cost and increasing scalability and flexibility, this provides the potential to increase distance by moving sensor reading dissemination into a long-distance gateway network infrastructure and enable interoperability by moving the management of heterogeneity into the more powerful devices provided by the gateway network. The gateway devices can be normal WiFi access points operating in the same frequency bands/channels as the sensor motes or smart boards endowed with WiFi, Ethernet, USB and Bluetooth interfaces such as the Alix boards from PCEngines [7] or computing platforms designed for software/cognitive radio applications such as proposed by [8]. Two different routing architectures can be derived from the use of our “gateway networking” solution. They are based on two different topologies that provide the potential to boost long distance and interoperable USN deployment in developing countries. These include a multi-star with mobile sink (MSMobiS) and a multi-star with meshed sink (MSMeshS) topologies. As illustrated by Figure 2, the MSMobiS deployment uses a topology where a set of sink devices is layered above islands of star networks of sensor motes. This topology depicted by Figure 2(a) does not require any multi-hop routing capability neither at the level of the islands of networks of sensor motes nor on the level of the network of gateway devices. However, it requires that the gateways to be endowed with GPRS or other wireless interfaces to convey the information wirelessly to processing places without local relay. Such deployment also lends itself to opportunistic data dissemination where data mules such as cars, humans, buses, *etc*. can be used in the gateway network to collect and store data in the gateways and fetch the data upon contact opportunities to forward this data for further processing, as suggested by the scenario of Figure 2(a) where a cellphone moving from positions 1 to 7 is used to upload the information collected from the sinks to the Internet. In a MSMeshS architecture, a mesh network of gateway devices is layered above islands of star networks of sensor motes as depicted by Figure 2(b). In such a network, multi-hop routing is implemented only at the level of the gateway network using protocols such as OSLR, BATMAN, AODV, *etc*. In this deployment, not all the gateway devices need to convey the information to the processing place. As depicted by Figure 2, sensor interoperability by combining, for example, normal sensors and mobiles used as sensors can be enabled by “gateway networking”. In both networking situations, The “gateway networking” model proposed in this paper is in agreement with the emergence of WiFi enabled sensor infrastructures that build upon the 802.11 family of protocols to achieve sensor reading dissemination as replacement for the energy-poor WPAN family of 802.15.4 protocols.

### 3.2. Opportunistic Data Dissemination

While Delay Tolerant Dissemination of data considers an Internet infrastructure where the network topologies are assumed to be known but with some links between gateways being available for only a given amount of times, opportunistic networking does not assume a priori knowledge of the network topology and computes routes “on-the-fly” by exploiting local knowledge at each hop to decide which is the best next hop among a node’s current neighbours to forward its data packets to in order to reach the eventual packet destination. In opportunistic networking, each single node acts as a gateway that can (1) forward its sensor readings when there is a node in its transmission range suitable for that transmission and (2) stores its data on local storage for a future contact opportunity for forwarding its data. Building upon the opportunistic networking model, we consider a “Drop-and-Fetch (DaF)” model where the gateways of a USN are considered as multi-technology mail boxes which are endowed with network selection capabilities allowing sensor readings to (1) be dropped into a gateway using a given protocol (WiFi, Bluetooth, GPRS/GSM) (2) be stored in waiting for a contact opportunity and (3) be fetched upon a contact opportunity by using the same or another technology depending on availability, time-of-the-day, quality of service provided by the network and type of sensor readings. The DaF paradigm is implemented in the access networking sublayers of our USN4D architecture by having a Mule-aware sublayer that can use different types of communication vehicles (cars, buses, trains, human travellers, *etc*.) to fetch data from the lower gateway sublayer and drop this data into the higher gateway sublayer where they are stored in waiting for a contact opportunity for further processing. Similarly, USN management information such as sensor queries or routing updates can be dropped into these vehicles from the higher gateway sublayer and opportunistically fetched by the lower gateway sublayer when specific data readings are required from the sensor networking layer. This is depicted by the three USN Access Networking sublayers in Figure 1(b) that abstract the opportunistic data dissemination by having the lower gateway sublayer managing the information to be dropped/fetched to/from the SNs, the Mule-aware sublayer managing the data mules (communication vehicles) and the higher gateway sublayer dealing with all communication to/from the Internet through interaction with the USN middleware. As modern technology has allowed the proliferation of multi-interface communication devices at affordable price, in our deployment scenario, a DaF device will be endowed with with bluetooth, WiFi and UMTS capabilities to make smart decisions by dropping/fetching the data carried into/from one of the three networks depending on the availability of Access Point and network proximity.

### 3.3. A Localised Middleware Model

A USN is an heterogeneous environment that requires a software layer often referred to as *middleware* that resides between programs, operating systems, hardware and communication platforms to let the different applications and systems work together. The main functions of such a middleware are threefold. Firstly, it aims at hiding the underlying complexity of the environment. Secondly, it helps in insulating the applications from explicit protocol handling, disjoint memories, data replication, network faults, and parallelism. Lastly, such a middleware will hide the heterogeneity of computer architectures, operating systems, programming languages, and networking technologies to facilitate application programming and management. As assumed by [9], this is done through easing the transformation of markup languages, delivery of content and data, recognition of protocols and devices, incorporation of and routing of business logic through enterprise systems and the adaptation of data formats for compatibility with multiple databases. In USN middleware approaches, how the network is abstracted is an important parameter that determines how the user wanting access to the network’s data will interact with the USN. The middleware proposed in this section is based on the Database abstraction model widely deployed as one of the implementation models for the commonly proposed middleware approaches for USNs. It includes (1) a data acquisition sublayer where raw data captured from sensor/RFID is captured into databases; and (2) an information adaptation layer where the stored information is translated into human readable language (e.g., voltage to numeric values) and localisation processes such as text-to-voice processing are performed to adapt to local constraints. Such localisation may be useful in the regions of the developing world where USN4D based CSNs are deployed for low literacy populations or where localisation to local languages and customs are a key requirement.

## 4. The WaspNet Development Platform

**Figure 3.**
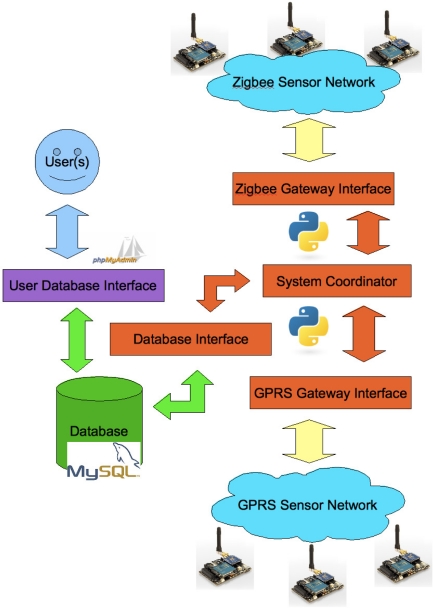
The WaspNet Development Platform.

As proposed in its experimental phase, WaspNet has been designed to use two different mote configurations. In both configurations, Waspmote main board is used in conjunction with a environmental board carrying different types of sensors and GPS board while information dissemination is achieved using either (1) a GPRS module and a Telit GC864 GSM modem interface to mobile network when using the GRPS mode or (2) a Waspmote XBeePro module and a Waspmote XBeePro gateway when operating in the ZigBee mode, as depicted by Figure 3. Both configurations use a SD Card to store readings if either the GSM or Zigbee networks are not available at the time when the reading is taken. Then, when the presence of the respective data network is detected, all the outstanding stored readings are uploaded to the WaspNet gateway.

Figure 3 depicts the main components of our USN platform and how they fitted in a WaspNet Testbed used in the “First Workshop on Wireless Sensor Networks and their applications to environment monitoring”, the first of a series of workshops organised on the African continent, held at the university of Cape Town in March 2010 [10]. The orange parts of the diagram represent the Python code. The green obviously represents MySQL and SQL links. The light yellow represents the lower level interfaces to the gateway interfaces (in both of these cases using serial links). The blue clouds represent the sensor networks. The approach is an attempt at a database abstraction, in which the sensor network is abstracted as a database of structured data for the end user. In order to achieve this, an actual relational database (MySQL) was used. The function of middleware software, which ran solely on the host system connected to the base station(s) of the sensor network, was to translate the data received from the sensor, and to insert the received data into the database. In order to achieve so, this received data was wrapped into Reading Objects, which themselves are classes based around a Python Dictionary primitive type, and passed from the base station interface module to the Database Interface by the Control Script. Thus all of the data received from the sensors in the network are inserted into a relational database of a general structure, allowing for the data to be extracted from the database, localised and analysed at a later point. While the middleware software has so far been run on Ubuntu Linux, it can be adapted to run under any operating system which supports Python and MySQL.

### 4.1. The Middleware Prototype Used by the Platform

**Figure 4.**
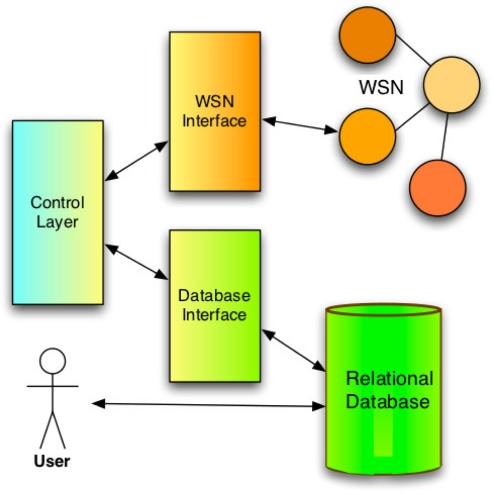
The WaspNet Middleware Components.

As depicted by Figure 4, the WaspNet middleware prototype includes the following components:

- *User abstraction* of the service that requires data from the WSN. It queries the WSN using the widely used Standard Query Language (SQL) and receives responses in the standard SQL format
- *Control/Interface Layer* that monitors the response from the relational database to the user. Trigger conditions can thus be set to be activated upon certain conditions and perform tasks. For example, when the timestamp of the data requested is too far in the past from the current time, the layer would in this case update the data in the database using the data from the WSN interface. Hence, this layer also can interact with the WSN interface, and use it to update the relational Database.
- *WSN Interface* to the actual Wireless Sensor Network that transform sensor readings in terms of voltage values into human friendly values. Depending on the WSN and the user requests and WSN responses, different formats are used for the sensor readings.
- *Relational Database* is the virtual version of the USN stored as a database. The structure of the database is dependent on the projected most common requests for data.

As implemented in the WaspNet Testbed, the model presented by Figure 3 includes the following open source software components implemented in Python and PHP:

- WSN Interface programmed in Python in order to communicate with the Waspmote Gateway over a USB connection. Essentially it contained a method for requesting readings from the sensors and a method for reading the responses from the sensors. The gateway also wrapped the data into Reading Objects.
- Readings are the raw data from the Wireless Sensors wrapped into Python Reading Objects, which contain all of the data sensed, along with information such as the Sensor Id and Network Id.
- Control Layer programmed in Python is a script that instructed the USN Interface to take readings and wrap them as Reading Objects. It then passed those Reading Objects to the Database Interface. The data reading procedure was time based.
- Database Interface programmed in Python, this interface interpreted the Reading Objects and inserted them into the relational Database used, in the decided upon structure.
- Relational Database is a standard MySQL database.
- User is the popular phpmyadmin database administration software used to interact with the relational database.

**Figure 5.**
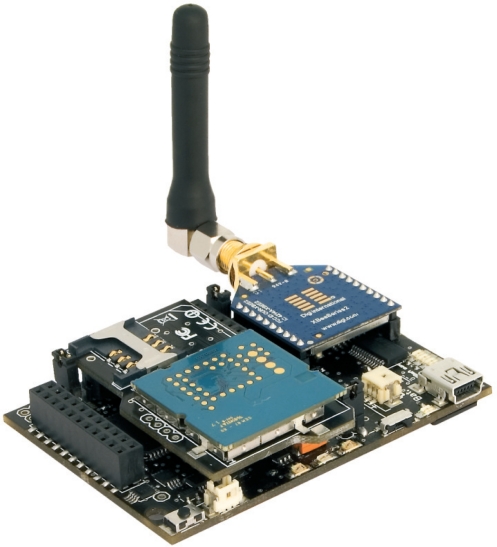
A Waspmote Device (courtesy of Libelium).

As depiected by Figure 5, WaspNet uses Waspmotes; a new generation of wireless sensor motes which have been recently released by Libelium [11]. They are built around an open software and hardware architecture using XBee transceivers [12,13] which provide several advantages in terms of multiplicity of operating power, protocols, and operating frequencies as depicted by the XBee features in Table 1.

### 4.2. WaspNet Motes and Gateways

Other characteristics include (1) minimum power consumption of the order of 0.7 *μA* in the Hibernate mode; (2) flexible architecture allowing extra sensors (such as gas, agriculture or physical events) to be easily installed in a modular way; (3) the provision of GPS, GPRS and SD card on board and (4) Xbee transceivers which are equipped with SMA antenna connectors so that an external antenna can be used. Furthermore, Waspmotes are powered with a lithium battery which can be recharged through a especially dedicated socket for the solar panel; this option is especially interesting for deployments in Developing Countries where power supply is not stable. We have shown in [14] that the XBee-868, XBee-900 and XBee-XSC transceivers series provide the potential to achieve longer distance deployments because of their lower frequencies used. However, the XBee-802.15.4 and ZigBee series operating in the 2.4 GHz ISM band have been adopted for our development platform since they meet more of the requirements of USN4D. These include the inter-operability with similar devices from different vendors and the possibility of building multi-layer USNs using Waspmotes and gateways operating in the same frequency band.

### 4.3. Opportunistic Data Dissemination

Besides using ZigBee communication, wireless sensor nodes (or motes, as they are commonly called) can use different other means of radio communication to send data to the gateway where measured data is stored. Several motes can be equipped with external GPRS modules and use the ubiquitous GSM network to act like mobile devices sending data either as SMS or with a GPRS data connection to upload this data to a web server. Building upon its modular design, the Waspmote by Libelium [11] is one example of mote platform than can use a GPRS module without any extra effort. As stated earlier, waspmotes can be equipped with different 802.15.4/ZigBee transceivers, but can also host a GPRS module to enable sending and receiving SMS, making and receiving calls and connecting to the GPRS network to transfer data. This has enabled the implementation of the DaF model into WaspNet by having both ZigBee and GPRS communication being based on Dropping data on local SD cards when communication is impossible and Fetching the information when a window of opportunity is offered by the presence of either a Zigbee gateway or the neighbourhood of a GRPS gateway with good signal strength.

### 4.4. Long Distance WaspNet Networks (LDWSN)

While most long distance wireless deployments have been focussed on the WiFi technology by fine-tuning the MAC protocol [15–17], to the best of our knowledge, long distance WSN deployment has been demonstrated only in the Waspmote family of sensor networks. Some of the works that have been done on long distance point to point sensor links include [18,19] and [20]. While the work presented in [18] proposed a sensor network in Australia where the range of a mote was extended up to 300 m, in [19] a switched beam directional antenna operating in the 2.4 GHz ISM band was used outdoors to extend the communication range from 140 m to more than 350 m. A long-range *ad-hoc* wireless sensor network is presented in [20]. In the proposal, a radio propagation model is used to enhance the range of wireless nodes to reach distances of up to 10 km. This was achieved by using non-directional antennae by having the radio transceiver of the Berkeley Mote replaced with a lower frequency and higher power unit operating in the 40.66–41.00 MHz frequency band with a maximum power of 1 W EIRP. Waspmote achieves much longer range compared to the hundred meters range limitation of many of the existing sensor technologies. The main differences between Waspmote and these technologies in order to get Long Distance USN (LDUSN) are (1) higher sensibility; (2) higher tx power; (3) the use of an external connector for the antenna (SMA) allowing the connection of antennas with a higher gain and with the correct polarisation; and (4) the use of lower frequency transceivers implemented by the XBee-868, XBee-900, and XBee-XSC family of Waspmote devices. It should also be observed that as the frequency plays a capital role in long range deployment by allowing longer range under low frequencies, WiFi operating only in the 2.4 GHz band cannot compete with the lower frequency transceivers of the Waspmote platform.

### 4.5. Readiness for Field Deployment

#### 4.5.1. Some Projects in a Training Class

To assess the readiness for field deployment of the WaspNet platform, we developed some scenarios and implemented these scenarios as illustrative Waspmote applications during the “First Workshop on wireless sensor networks with application to environment monitoring” held in Cape Town in March 2010 [10]. The following projects were given and successfully executed by the participants as tasks related to the applications of sensors as mobile devices.

- I want to monitor a room’s temperature every 5 min. I want to receive the temperature via SMS and a special alert if it is greater than 20 °C.
- It has been reported that in some developing countries, the theft of containers during transportation from the shore to the city is common practice. I own a container and want to check that if it moves, it follows a predefined itinerary. If during transportation it moves from its itinerary, it means that it’s being stolen. Send an SMS with its GPS position if there is an acceleration and its position is out of a predefined range.
- I want to check if a motor is behaving properly. I should measure its acceleration four times per second and send the information via SMS or another wireless method to a PC.
- I want to measure the pollution in Cape Town. Read data every 30 seconds from a Gas Sensor board and store it on the SD card with date, time and position while sending SMSs to a given number when pollution reaches given threshold values.

These tasks successfully executed by the participants using the waspmote platform reveal how sensors can be used as mobile devices sending and receiving SMS.

#### 4.5.2. Long Distance Deployment

**Figure 6.**
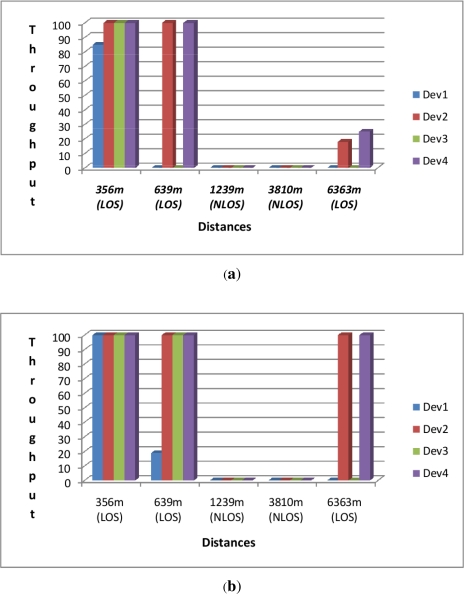
Waspmote throughput on Long Distances. (**a**) Throughput at 2 dBi (in %); (**b**) Throughput at 5 dBi (in %).

We also considered experimental results obtained over a period of 3 days using XBee Waspmote transceivers operating in the 2.4 GHz band. We wanted to check how far these different transceivers could go in terms of range and throughput when sending 100 packets of 90 Bytes each from a source node to a distant destination in both Line of Sight (LOS) and Non-Line-of-Sight (NLOS) for distances ranging from 356 m to 6,363 m. The results are summarised in Figure 6 for the 4 waspmote transceivers that use the 2.4 G frequency band as this is the ISM band which is license free in most of the developing countries. These transceivers are referred to as Dev1, Dev2, Dev3, and Dev4 in Table 2.

These results (1) show that Waspmotes are the first generation of sensor motes that can achieve long distance USN links of up to 6 km and (2) agree with the common knowledge that reveal the increase of throughput with the sensitivity (e.g., 5 dBi gives better throughput compared to 2 dBi) and the transmission power of the transceiver (802.15.4-Pro and ZigBee-Pro series lead to higher throughput because of their higher power).

#### 4.5.3. Encryption and Security

**Figure 7.**
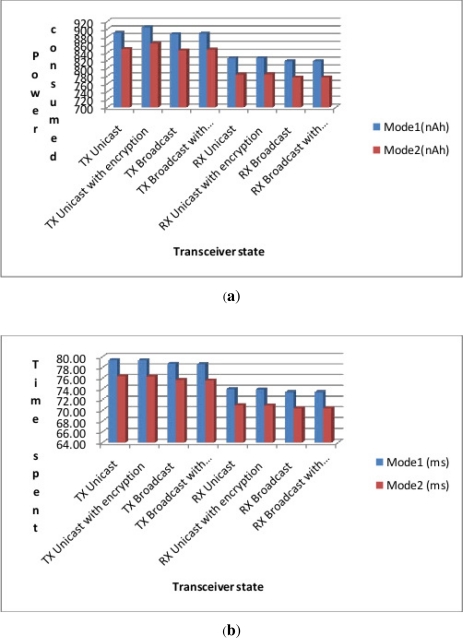
Waspmote Power and Time under Encryption. (**a**) Energy consumed in a mode (in nAh); (**b**) Time spent in a mode (in ms).

During the experiments we also made some measurements to assess the impact of encryption and its related security benefits on the power consumption of the Waspmotes. We used four different types of transmissions: (1) Unicast without encryption (2) Unicast with encryption (3) Broadcast without encryption and (4) Broadcast with encryption. Note that in these experiments, we measured the time and energy consumption from the OFF to ON mode referred to as “Mode 1” and from the SLEEP to the ON mode called “Mode 2” with the objective of evaluating what is the best energy saving mode for a possible synchronisation algorithm. In case of a unicast transmission the protocols waits for an ACK signal, while in case of broadcast there is no ACK. However, in broadcast mode each packet is always sent three times. The encryption (AES 128b) does not add any consumption due to the fact that it is performed using specific hardware circuits included in the XBee card and not in the software layer. The results summarised in Figure 7 reveal that (1) Mode 1 is more power consuming than Mode 2, (2) encryption and its related security benefit does not come at the price of a high cost in terms of power consumption, and (3) the time spent by Waspmotes in Mode 1 is higher than than the time spent in Mode 2. This suggests the implementation of security strategies that favour the second mode to save on energy consumption and processing time.

#### 4.5.4. Application to Pollution Monitoring in the City of Cape Town

Resulting from the presence of contaminants or pollutant substances produced by vehicle emissions, industrial emissions, and volatile organic compounds, air pollution is a threat to both humans and environment which, according to the World Health Organisation, kills 2.4 million each year. While being widely recognised as a health threat causing serious problems like asthma, cancer, and heart disease in the developed world, pollution is still an uncontrolled issue in developing countries where millions of people die each year from the effects of air and water pollution. As currently implemented, pollution monitoring is based on air and water quality monitors sparsely deployed at relatively small number of fixed locations by governmental organisations. This creates a visibility gap that needs to be addressed through complementary technologies, systems and strategies. As a first step towards providing environmental data to cities, WaspNet was designed to build a Community Sensor Network (CSN) that in its first phase would be (1) collecting pollution data using air, water and camera sensors, (2) analysing these data, (3) modelling pollution in cities starting by Cape Town, and (4) providing awareness to citizens, official organisations, Non Governmental Organisations and private organisations.

### 4.6. The Gas Board and Experimental Setting

**Figure 8.**
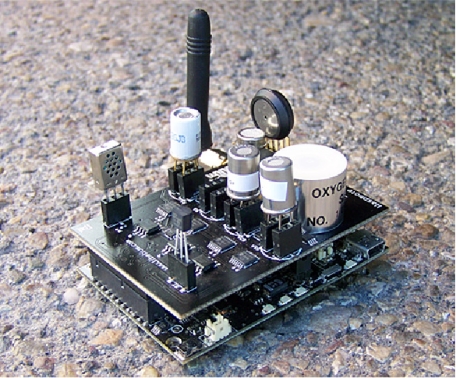
The Gas Sensor Board (courtesy of libelium).

The Waspmote gases sensor board depicted by Figure 8 has been designed to monitor environmental parameters such as temperature, humidity, atmospheric pressure and 11 different types of gases. It allows the inclusion of 6 gases sensors at any one time, the regulation of their power through a system of solid state switches and the amplification of the output signal through a controllable amplifier. The gases which can be monitored are (1) Carbon Monoxide CO; (2) Carbon Dioxide CO_2_; (3) Molecular Oxygen O_2_; (4) Methane CH_4_; (5) Molecular Hydrogen H_2_; (6) Ammonia NH_3_; (7) Isobutane C_4_H_10_; (8) Ethanol CH_3_CH_2_OH; (9) Toluene C_6_H_5_CH_3_; (10) Hydrogen Sulphide H_2_S; and (11) Nitrogen Dioxide NO_2_.

To improve the accuracy of these sensors, a calibration procedure can be used, in effect creating a clean air baseline against which gas presence levels can be measured. This calibration procedure consists of taking a number of readings in clean air, and averaging the raw resistance values recorded. Building upon the WaspNet Testbed, we conducted sets of experiments to assess the readiness for field deployment of the system in terms of (1) pollution level measurement and mapping and (2) information dissemination using both ZigBee and GPRS connectivity when measuring pollutant levels in the city of Cape Town, and publishing the results as pollution maps using Google Maps. We conducted experimental trials using both WaspNet mote configurations, with both devices being carried in a car driving in defined target areas. We conducted three sets of trials during the experiment: one on the 23 July 2010, the second on 24 September 2010, and the third on 29 September 2010. For the first set of experiments conducted on the 23 July, we used as starting point (as the source for the calibration) a house located in leafy and low-density population suburb called Fernwood, near the National Botanical Gardens. All subsequent readings were relative to the air pollution levels at this house. The second and third sets of experimental trials were conducted on 24 and 29 September 2010, both using as starting point the higher campus of the University of Cape Town as the calibration point. The first and third trials targeted primarily the northern half of the Southern suburbs and Northern suburbs of Cape Town, while the second trail targeted the southern half of the Southern suburbs. The main results expected from our experimental setting were expressed in terms of readiness for field deployment and pollution mapping and publishing by (1) producing a pollution map with tagged locations of pollution thresholds using Google Maps based upon readings taken from all the experimental trials and (2) proposing graphs of the relative levels of different pollutants to reveal their concentration. The readiness for field deployment was measured by (1) the battery lifetime in both GRPS and ZigBee modes and (2) the packet loss in both modes.

### 4.7. Pollution Mapping and Publishing

**Figure 9.**
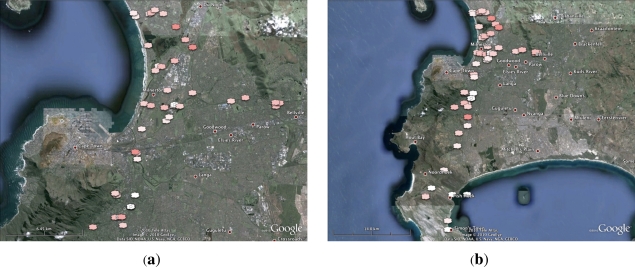
Pollution Mapping. (**a**) Pollution Map (First Day); (**b**) Pollution Map (Second Day).

To measure the relative levels of pollutants, we used a cloud representation where redder clouds represent higher pollution reading at that point. The pollution readings are recorded by the mote as a voltage level measured across the pollution sensor at that particular point. This is then used to calculate the resistance of the sensor, which is measured relative to calibrated “clean air” value. Two types of clouds are used: those without circles representing the pollutant levels taken in Zigbee mode and the ones with circles within them which present the pollutant levels taken in GPRS mode. The readings were averaged at a particular point if multiple readings were taken at that geographic location and the time label considered was the one of the last reading taken at that point. This provided images that give an overview of the results by spotting general trends with regards to the sensors. Looking at the pollution map depicted by Figure 9(a,b), one should note that the sensor responds to the levels of the whole group of the gases, and so is a composite value of the various gas concentrations present. While this does not allow for accurate measurement of the level of a particular gas, it does give an effective neighbourhood which the various gas levels fall within, and thus an indication of the level of air pollution.

Using both map plots, it does seem to make sense that the Northern Suburbs of Cape Town is more polluted than the Southern Suburbs, especially given the presence of an oil refinery in the area.

### 4.8. Readiness for Field Deployment

**Figure 10.**
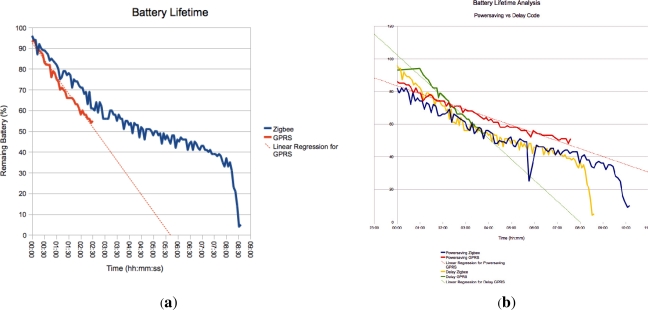
Battery Lifetime.

Figure 10 reveals the battery lifetime in both ZigBee and GPRS modes. As shown by the Figure and in agreement with the datasheet, the GPRS module was not working after the battery dropped below 55%, causing the GPRS modem to shutdown. This is illustrative of the need for further prototyping and performance testing, as was the fact that the data was not saved to that module’s SD card (the other module’s SD card worked fine) in the first trial. In the second trial, there was a problem with the GPS module used with the GPRS mote, not allowing for locations of the readings to be recorded. However, by the third trial, the various issues were worked out, and the devices performed well.

**Figure 11.**
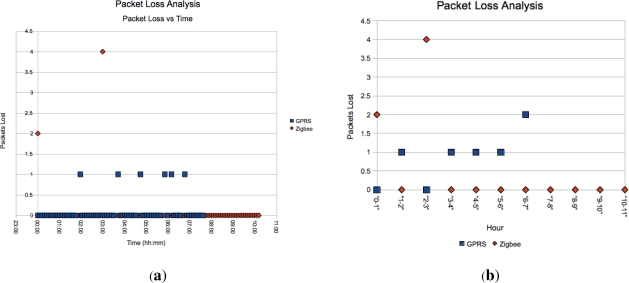
Packet Loss.

The 10% packet loss calculated using a sequence numbering scheme in the packets for the GPRS sensor, which is quite high, is in agreement with recent events in South Africa, where the mobile operators were fined heavily last year because 1 in 20 SMSs were not reaching their destination. This finding suggests moving to actually using GPRS as opposed to SMSs, due the exponentially lower cost of GPRS data links *versus* SMS messages and reliability mechanisms built into Internet protocols. However, the issue of availability of GPRS *versus* GSM in the target area must then be considered. The packet loss for the Zigbee sensor was also 10%. For the packet loss, Figure 11(a) shows where the packet loss occurred while Figure 11(b) summarises the instances into hourly bins. The distribution of the dropped packets for the GPRS is quite widely spread, as is to be expected, while the Zigbee is concentrated at the beginning and one anomalous instance.

## 5. Conclusions and Future Work

Motivated by the need to deploy USN in developing countries, this paper presents a new USN architecture referred to USN4D. USN4D extends the ITU USN model to account for LDUSN deployments and localised information dissemination in terms of language and technology availability. We present the WaspNet as one of the first USN platforms that achieves Long Distance USN (LDUSN) deployment, security, opportunistic data dissemination and meets different other constraints of the developing World such as the necessity of using least cost off-the-shelf technology for cost reduction and maintenance. Preliminary experimental results reveal the potential offered by the WaspNet platform adopted for our USN implementation. Furthermore, building upon the need to build community sensor networks providing services to cities, this paper shows how the first WaspNet platform was successfully used to monitor air pollution in the city of Cape Town. As presented, WaspNet integrates the main ideas behind a community sensor networking deployment aiming at launching sensors in the environment to build environmental maps and promote public participation in the fight against air pollution. Our experimental setting reveals that (1) pollution map can be built using off-the-shelf sensor equipment, (2) both Zigbee and GPRS can be used as information dissemination protocols for the sensed data, (3) this can be done with low packet loss, and (4) the experimental results may be published using web services such as Google Maps.

It should be emphasized that the experiments undertaken in this paper were mainly part of a proof of concept, assessing the field-readiness of the WaspNet platform in terms of pollution measuring and dissemination by comparing the performance of the direct transmit GSM transmission communication *versus* the store-and-burst transmit Zigbee communication. These experiments hence form part of the iterative development of on-going research which is being extended in several different directions:

- In collaboration with the city of Cape Town and the University of Cape Town SHAWCO NGO, a larger number of sensors will be used in a near future to cover a greater area, using both static sensors located in some fixed locations by the city such as some specific traffic light locations and mobile sensors mounted on buses of the SHAWCO NGO.
- The gas sensors used will be calibrated, and correction functions will be developed.
- The overall reliability of the system is being improved, with the development of robust enclosures that will allow for greater consistency across the sensor platforms.
- Evaluation of opportunistic transmission in the presence of different types of gateways.
- The running of the experiments to evaluate the impact of daily climate variations and road traffic fluctuations on pollution.
- The design of an intelligent and user-friendly interface that allows users to extract data from the database without using phpMyAdmin and find hidden patterns in the environmental data collected to enable reasoning and decision making.

## Figures and Tables

**Table 1.** Waspmote Transceivers.

**Model**	**Protocol**	**Frequency**	**TX power**	**Sensitivity**
XBee-802.15.4	802.15.4	2.4 GHz	1 mW	−92 dB
XBee-802.15.4-Pro	802.15.4	2.4 GHz	63 mW	−100 dB
XBee-ZB	ZigBee-Pro	2.4 GHz	2 mW	−96 dB
XBee-ZB-Pro	Zigbee-Pro	2.4 GHz	50 mW	−102 dB
XBee-868	RF	868 MHz	315 mW	−112 dB
XBee-900	RF	900 MHz	50 mW	−100 dB
XBee-XSC	RF	900 MHz	100 mW	−106 dB

**Table 2.** Waspmote Transceiver Features and Performance.

**XBee Features**	**Dev1**	**Dev2**	**Dev3**	**Dev4**

Protocol	802.15.4	802.15.4	ZigBee-Pro	ZigBee-Pro
Frequency (Hz)	2.4 G	2.4 G	2.4 G	2.4 G
TX power (mW)	1	63	2	50
Sensitivity(*−*dBm)	92	100	96	102
